# Kirchhoff Law Johnson noise key generation for secure decentralized identifiers

**DOI:** 10.1038/s41598-025-34403-7

**Published:** 2026-01-08

**Authors:** Kamalesh Mohanasundar, Sarah A. Flanery, Srujan Kotikela, Christiana Chamon

**Affiliations:** 1 Department of Computer Science, University of Texas at Dallas, Richardson, 75080 TX USA; 2 Department of Electrical and Computer Engineering, Texas A&M University, College Station, 77843 TX USA; 3 Department of Information and Operations Management, Texas A&M University, College Station, TX 77843 USA; 4 Department of Electrical and Computer Engineering, Virginia Tech, Blacksburg, 24060 VA USA

**Keywords:** Engineering, Physics

## Abstract

This paper experimentally integrates an existing Kirchhoff–Law–Johnson–Noise (KLJN) physical key exchange scheme as a source of truly random keys for decentralized identifiers (DIDs). Web 3.0 is driven by secure keys, typically represented in hexadecimal, that are pseudo-randomly generated by an initialization vector and complex computational algorithms. We demonstrate that the statistical physical KLJN scheme eliminates the additional computational power by naturally generating physically random binary keys to drive the creation of DIDs that are appended to an Ethereum blockchain.

## Introduction

### Link to web 3.0

1Web 3.0 currently exists with the purpose of bringing back ownership to its users^1,2,4^. To accomplish this, it takes a decentralized approach using blockchain technology^5^. Similar to a linked list, blockchains consist of blocks appended to a preexisting list. Each block contains information about the previous and preceding block. However, no previous blocks can be deleted or altered^6^.

Information security in the present day is highly centralized; all data is controlled by singular entities such as the protocols within SSL and TLS, and users have to trust third parties to verify that their data is encrypted^7,8^. For example, social media companies such as X (formerly known as Twitter), Facebook, and Instagram are all operated by a centralized power: the admin assigned by each company. This system of storing information is based on Web 2.0. The basis of Web 2.0 is that a central power ensures the safety and accuracy of information online and decides what is distributed to its users. These administrators control what information is distributed to its users and which must be hidden or deleted from the platform. For these reasons, this has the potential to be a gateway to corruption, as the basis of the population’s access to information are influenced by personal biases^9^.

Web 3.0, due to the nature of its data structure, eliminates this problem entirely by removing the centralized power and using other methods of verification to determine the accuracy of information on the internet. The obstacle encountered when trying to incorporate Web 3.0 into education is the centralized nature of learning. In modern society, the validity of an academic credential is established by the institution itself rather than defining individual skills learned. In Web 2.0, the user does not have any ownership of the information that they post online. However, Web 3.0 resolves this issue. In the present paper, we demonstrate that the decentralized elements of Web 3.0 can be utilized in order to ensure that the accomplishments in education remain assigned to the user.

### Decentralized identity

**Fig. 1:**
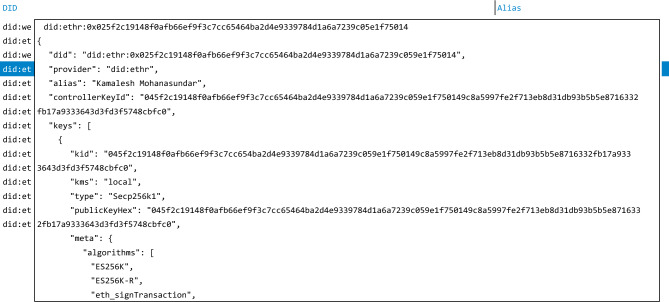
Example DID document structure with key fields: identifier, controller, public key, and algorithm metadata^2^.

Decentralized identity has emerged as a foundational component of Web 3.0 infrastructure, enabling users to control their own digital credentials without reliance on centralized identity providers^10^. Figure 1 shows an example of a DID document. The W3C DID Core specification defines the standard for creating and resolving decentralized identifiers (DIDs), allowing individuals and organizations to cryptographically prove ownership and control of their identities on distributed ledgers^11,12^.

Recent advances in DID systems have focused on enhancing security, scalability, and interoperability. Xian et al. provide a comprehensive survey of decentralized identity management systems, covering architectures, consensus mechanisms, and privacy-preserving techniques across multiple blockchain platforms^13^. Li et al. introduce DisIMS, a fully distributed identity management system that leverages blockchain for transparent and auditable identity operations^14^. Deng et al. propose FutureDID, a fully decentralized identity framework with multi-party verification to prevent single points of compromise^15^. Zheng et al. present IDEA-DAC, an advanced credential system using zero-knowledge JSON proofs to enhance privacy while maintaining auditability^16^.

However, a critical gap remains: all existing DID systems, including those cited above, depend on pseudorandom or deterministically generated cryptographic keys for the underlying identifiers. These keys are typically produced by computationally intensive algorithms such as elliptic curve cryptography (ECC), whose security relies on the assumption that certain mathematical problems are computationally hard–an assumption that may be threatened by advances in quantum computing or algorithmic breakthroughs . Furthermore, the randomness of these keys depends entirely on the quality and unpredictability of the seed value supplied to the pseudorandom number generator (PRNG), a dependency that has been exploited in known RNG attacks^17–28^.

Our work addresses this gap by demonstrating that DID key generation can instead be grounded in physical randomness derived from thermal noise, leveraging the unconditional security guarantees of the Kirchhoff–Law–Johnson–Noise (KLJN) scheme. Unlike the systems surveyed above, the KLJN-based approach is not dependent on computational hardness assumptions or algorithmic RNG security; rather, it derives its security from fundamental physical principles (the second law of thermodynamics). By integrating KLJN key generation directly into a DID framework (Veramo), we show that DIDs can be established with information-theoretic security, independent of the number of users and resistant to RNG-based attacks.

The DID keys are generated using the base encryption methods for Ethereum blockchains, i.e. a combination of public and private key encryption^29^. The public key can be generated using a public key hex and ES256 algorithms^30^. The private key is generated based on elliptic curve cryptography which is commonly used in Bitcoin^30,31^. However, the vulnerability with the pseudo-random generation is that if either of the keys are found, anyone can access the user. Additionally, these pseudo-random methods involve multiple computational steps, thus the amount of computational power increases accordingly. These results show that secure keys can be generated without computational overhead, i.e., the keys can be generated naturally. Unlike conventional deterministic or computational key generation methods commonly used in decentralized identity systems, which rely on algorithmic processes vulnerable to cryptographic and implementation attacks, our approach leverages the KLJN scheme to achieve true information-theoretic security^3,17–19,32–53,53–95^. Conventional DID key generation mechanisms have been shown to be susceptible to attacks leading to full impersonation, potential permanent loss of digital identity, and unauthorized access^96–109^. By exploiting the intrinsic randomness of thermal noise, the KLJN scheme eliminates these vulnerabilities, ensuring that secure keys can be generated independently of the number of users without relying on computational hardness assumptions. In the present paper, we explore how the statistical physical phenomena of the KLJN secure key exchange scheme can generate keys to support the Semantic Web protocols that drive DIDs.

### The KLJN scheme

The KLJN scheme is a statistical physical scheme that leverages the well-established Johnson-Nyquist noise phenomenon, which describes the thermal noise voltage generated across a resistor due to random electron motion^110^. This fundamental physical process provides the randomness source for the key exchange.

Key system parameters include resistor values, noise bandwidth, temperature control, and sampling rates. Resistor values are selected to maximize the distinguishability of valid states while minimizing information leak. The noise bandwidth determines the bit generation speed, balancing between throughput and noise averaging for secure communication. Temperature uniformity ensures consistent noise levels. Sampling rates must comply with Nyquist criteria to capture full noise information^111^. Careful hardware selection and calibration are necessary to maintain these parameters within secure operational margins.

**Fig. 2:**
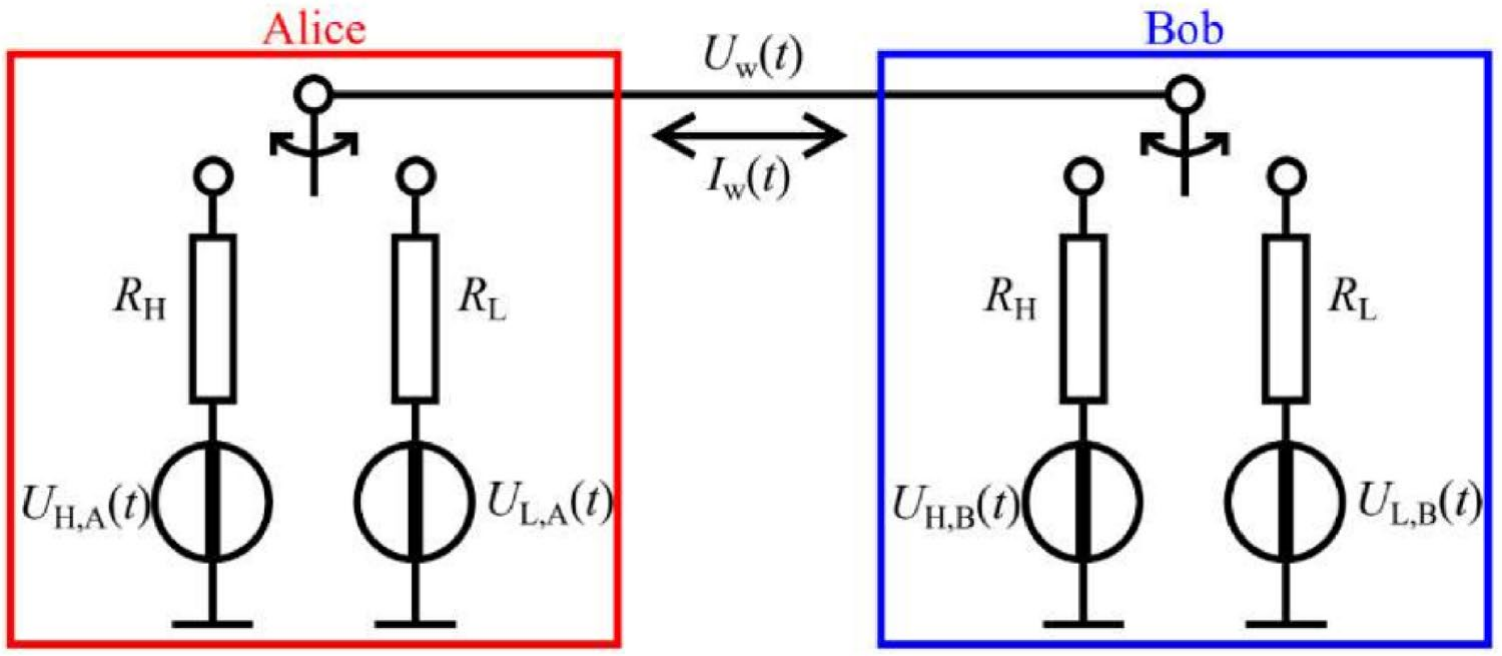
KLJN core elements: Alice and Bob connect via a shared wire, each randomly selecting resistor $R_{\textrm{H}}$ or $R_{\textrm{L}}$, and observing mean-square noise voltage $U_{\textrm{W}}(t)$ to exchange secure bits^17–19,37^.

Figure 2 illustrates the core of the KLJN scheme. Communicating parties Alice and Bob are connected by a publicly accessible wire, which has voltage and current, denoted by $U_{\textrm{w}}(t)$ and $I_{\textrm{w}}(t)$, respectively. Each communicating party has identical pairs of resistors $R_{\textrm{H}}$ and $R_{\textrm{L}}$ ($R_{\textrm{H}} > R_{\textrm{L}}$) and their respective artificial noise generators $U_{\textrm{H,A}}(t)$, $U_{\textrm{L,A}}(t)$, $U_{\textrm{H,B}}(t)$, and $U_{\textrm{L,B}}(t)$ (the former two belonging to Alice, the latter two belonging to Bob) that emulate thermal noise at a publicly-agreed-upon $T_{\textrm{eff}}$ (typically > $10^{15}$ K).

To generate a single bit, Alice and Bob begin the bit exchange period (BEP) by randomly selecting one of their resistors to connect to the wire, observing the instantaneous noise voltages over the wire, and taking the mean-square value of those noise voltages to assess the bit status. The mean-square voltage is given by the Johnson Formula,

1 $$\begin{aligned} U_{\textrm{w}}^2 = 4kT_{\textrm{eff}}R_{\textrm{p}}\Delta f_{\textrm{B}}, \end{aligned}$$

where *k* represents the Boltzmann constant ($1.38 \times 10^{-23}$ J/K), $T_{\textrm{eff}}$ represents the publicly-agreed-upon effective temperature, $R_{\textrm{p}}$ represents the parallel combination of Alice’s and Bob’s chosen resistors, given by

2 $$\begin{aligned} R_{\textrm{p}} = \frac{R_{\textrm{A}}R_{\textrm{B}}}{R_{\textrm{A}}+R_{\textrm{B}}} \end{aligned}$$

where $R_{\textrm{A}}$ represents Alice’s resistor choice and $R_{\textrm{B}}$ represents Bob’s resistor choice, and $\Delta f_{\textrm{B}}$ represents the noise bandwidth of the generators.

**Fig. 3:**
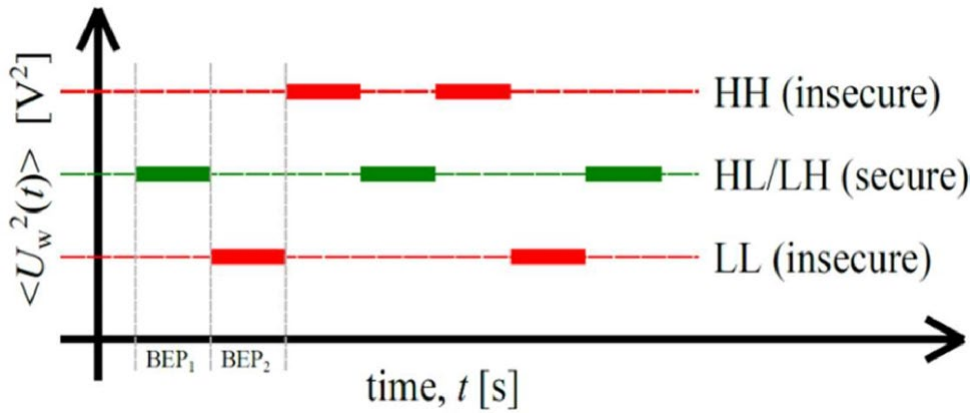
The three mean-square voltage levels. The HH and LL cases represent insecure situations because they form distinct mean-square voltages. The HL and LH cases represent secure bit exchange because Eve cannot distinguish between the corresponding two resistance situations (HL and LH). On the other hand, Alice and Bob can determine the resistance at the other end because they know their own connected resistance value^17–19,37^.

Four possible resistance combinations can be formed by Alice and Bob: HH, LL, LH, and HL. Using the Johnson Formula, these correspond to three mean-square voltages, as shown in Fig. 3. The HH and LL cases are insecure situations because they render a distinct mean-square voltage; these bits are discarded by Alice and Bob. The LH and HL cases, on the other hand, are secure situations because they render the exact same mean-square voltage, thus an adversary cannot differentiate between the two situations; however, Alice and Bob know which resistor they have chosen.

We typically represent the LH case as the bit value “0” and the HL as the bit value “1”^38,88^. Over the course of several BEPs, Alice and Bob generate a secure binary key, or random string of bits (see Section Decentralized Identity). All binary keys can be converted into a hexadecimal representation, thus we believe that a system that utilizes hex keys can be driven by converting the base of the original binary keys. In the present paper, we explore a proof of concept involving the utilization of the statistical physical phenomena of the KLJN scheme to provide secure keys to drive a DID credentialing system that we created^2^.

### Security analysis

CSPRNGs generate pseudorandom sequences deterministically from an initial seed. While widely used, their security fundamentally depends on the seed’s secrecy and unpredictability. If the seed or algorithm is compromised or weak, the pseudorandom outputs become predictable, enabling attacks such as seed recovery and RNG exploits^20–28^.

In contrast, the KLJN scheme derives its randomness from physical noise sources–specifically, thermal noise governed by the fluctuation dissipation theorem^112^. This approach provides true randomness that is not algorithmically reproducible, offering foundational information-theoretic security impervious to RNG-related attacks^17,19,37^. Additionally, KLJN’s reliance on simple hardware components avoids the computational overhead typical of CSPRNGs, making it especially suitable for resource-constrained environments.

Although the KLJN key exchange scheme is information-theoretically secure based on the second law of thermodynamics, practical implementations face challenges. These attacks include cable capacitance effects, current injection, transient response exploitation, and electromagnetic or thermal side-channel leakage. However, extensive research has proposed defense protocols and hardware design optimizations to mitigate these vulnerabilities^17–19,37–41,75–79,81,82,84–93,113–115^. For instance, cable capacitance effects can be neutralized via matched filtering and shielded cable design^81^, while current injection attacks are countered by real-time monitoring of current and voltage levels^79^. Our implementation aligns with these secure design principles, ensuring that the practical system retains the unconditional security characteristics of the ideal KLJN setup.

Our KLJN implementation builds upon extensive research demonstrating that both ideal and practical KLJN systems achieve unconditional security through tailored defense protocols. Practical attacks such as cable capacitance, current injection, and transient signal attacks have been studied thoroughly. Proposed countermeasures include balanced cable designs, real-time monitoring of current and voltage waveforms, and transient signal filtering to prevent leakage of information. Importantly, even in scenarios where a minor information leak might occur, privacy amplification techniques–shortening the raw key to a smaller, more secure key–can be applied to maintain unconditional security at the cost of reduced bit rate^53,116–123^. These combined strategies uphold the theoretical security foundations under practical non-ideal conditions, ensuring robust protection in real-world environments.

The primary scientific and engineering contribution of this work lies in addressing a critical security gap in decentralized identity systems: the reliance on insecure pseudorandom key generation mechanisms. While the KLJN key exchange scheme itself is established, its integration as a physical-layer entropy source for real-world DID protocols (e.g., Veramo) provides a novel, testable approach to achieving information-theoretic key security–previously unaddressed in DID systems. This integration showcases a methodology to reliably generate cryptographic keys grounded in fundamental physical randomness rather than deterministic computations, thereby reducing the attack surface for identity forgery and impersonation. Our experimental results validate this integration’s feasibility and highlight the potential for wider adoption of physical-layer security primitives in emerging decentralized identity systems. Hence, beyond a software integration, the work proposes and experimentally validates a new security primitive in DID that advances the field both scientifically and practically.

### Our impact

This work makes the following contributions:

- Introduces the KLJN scheme as a physical entropy source for generating DIDs in a decentralized framework.
- Demonstrates end-to-end integration of KLJN-generated keys with Veramo and Ethereum, showing that DID creation can be driven entirely by physical randomness instead of pseudorandom seeds.
- Provides statistical validation of KLJN-generated 256-bit keys using established test suites and compares qualitative performance characteristics with conventional CSPRNG and ECC-based methods.
- Discusses how KLJN-based key generation can reduce attack surfaces associated with seed compromise and RNG weaknesses in decentralized identity systems.

The rest of this paper is as follows. In the following section, we describe the methodology. Then, we demonstrate the KLJN-powered identifier and compare its performance to conventional cryptographic schemes. Finally, we conclude this paper.

## Methodology

Alice and Bob randomly select one of their resistors, $R_{\textrm{H}}$ or $R_{\textrm{L}}$, to connect to the wire (see Section The KLJN Scheme). If both communicating parties chose $R_{\textrm{H}}$ or $R_{\textrm{L}}$ (i.e. the HH or the LL case), we discard the bit; however, in the secure bit situations where Alice chose $R_{\textrm{H}}$ and Bob chose $R_{\textrm{L}}$ (the HL case), or where Alice chose $R_{\textrm{L}}$ and Bob chose $R_{\textrm{H}}$ (the LH case), we keep the bit.

Alice and Bob undergo as many BEPs as needed to produce a secure binary key of a fixed length *L*. We employ base conversion to convert the binary key to its respective hexadecimal representation, which contains $\frac{1}{4}L$ characters. We then use the resulting hex key to generate a DID for a single user and append the DID to an Ethereum blockchain.

## Demonstration

### Statistical key generation

#### Johnson noise emulation

2First, we numerically generated Gaussian band-limited white noise (GBLWN) in MATLAB, taking care to mitigate aliasing, improve Gaussianity, and reduce short-term bias. We created a long sequence of Gaussian samples using the built-in randn() function, and several independent realizations were averaged to obtain a single time series with improved statistical properties. This series was then transformed to the frequency domain by a fast Fourier transform (FFT), zero-padded to enforce Nyquist-compatible sampling and suppress aliasing, and finally brought back to the time domain via an inverse FFT. The real part of this inverse-transformed signal served as the band-limited, anti-aliased noise time series used in the simulations. The methodology follows the approach originally detailed in^3^, with parameter choices adjusted for the present work.

**Fig. 4:**
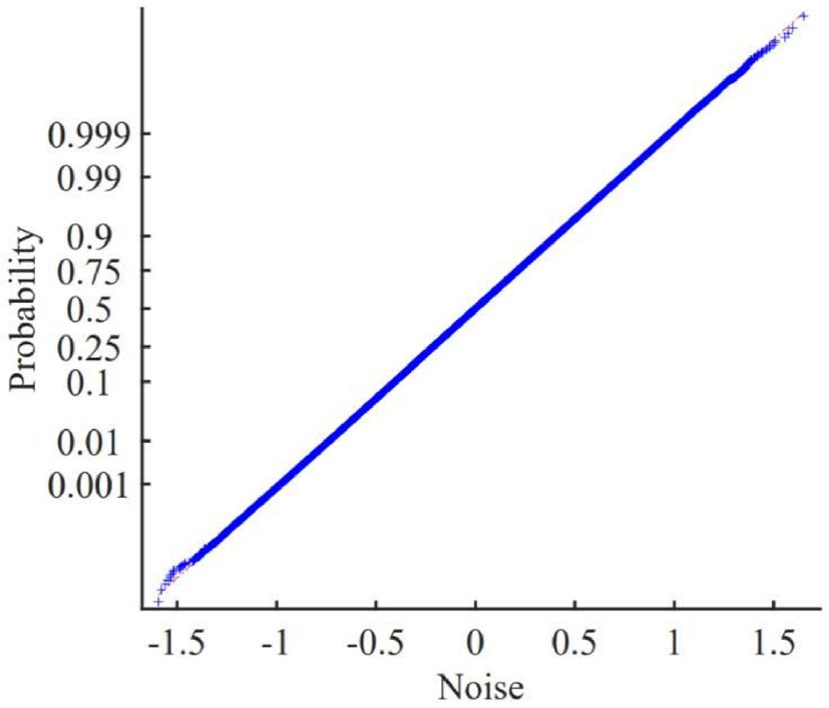
Normal-probability plot of the noise^17–19,37^. A straight line indicates a pure Gaussian distribution.

**Fig. 5:**
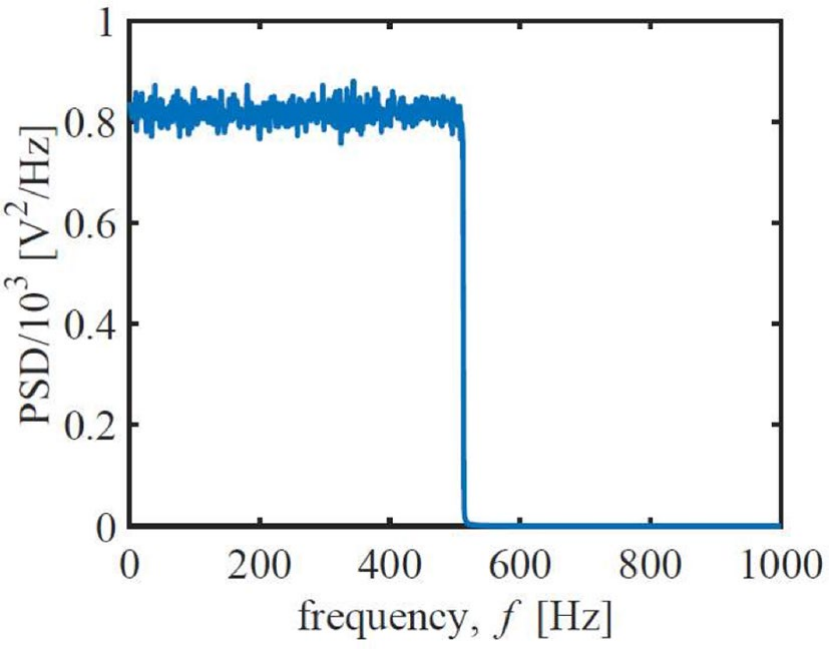
Power spectral density of the noise^17–19,37^. The bandwidth of the noise is 500 Hz, see (Equation (1)).

The probability plot of the generated noise is shown in Fig. 4, showing that the noise is Gaussian. Figure 5 demonstrates that the noise has a band-limited, white power density spectrum and that it is anti-aliased.

From the Nyquist Sampling Theorem,

3 $$\begin{aligned} \tau = \frac{1}{2\Delta f_{\textrm{B}}} \end{aligned}$$

where $\tau$ represents the time step, an $\Delta f_{\textrm{B}}$ of 500 Hz renders a time step of $10^{-3}$ seconds (i.e., a sampling frequency $f_s$ of 10 kHz).

The resulting normalized Gaussian noise was scaled to emulate Johnson noise for the target resistance, bandwidth, and effective temperature. In this study we chose $R_{\textrm{H}}=100$ k$\Omega$, $R_{\textrm{L}} = 10$ k$\Omega$, noise bandwidth $\Delta f_{\textrm{B}}=500$ Hz, and effective temperature $T_{\textrm{eff}} = 10^{18}$ K, consistent with established KLJN simulation practices.

**Fig. 6:**
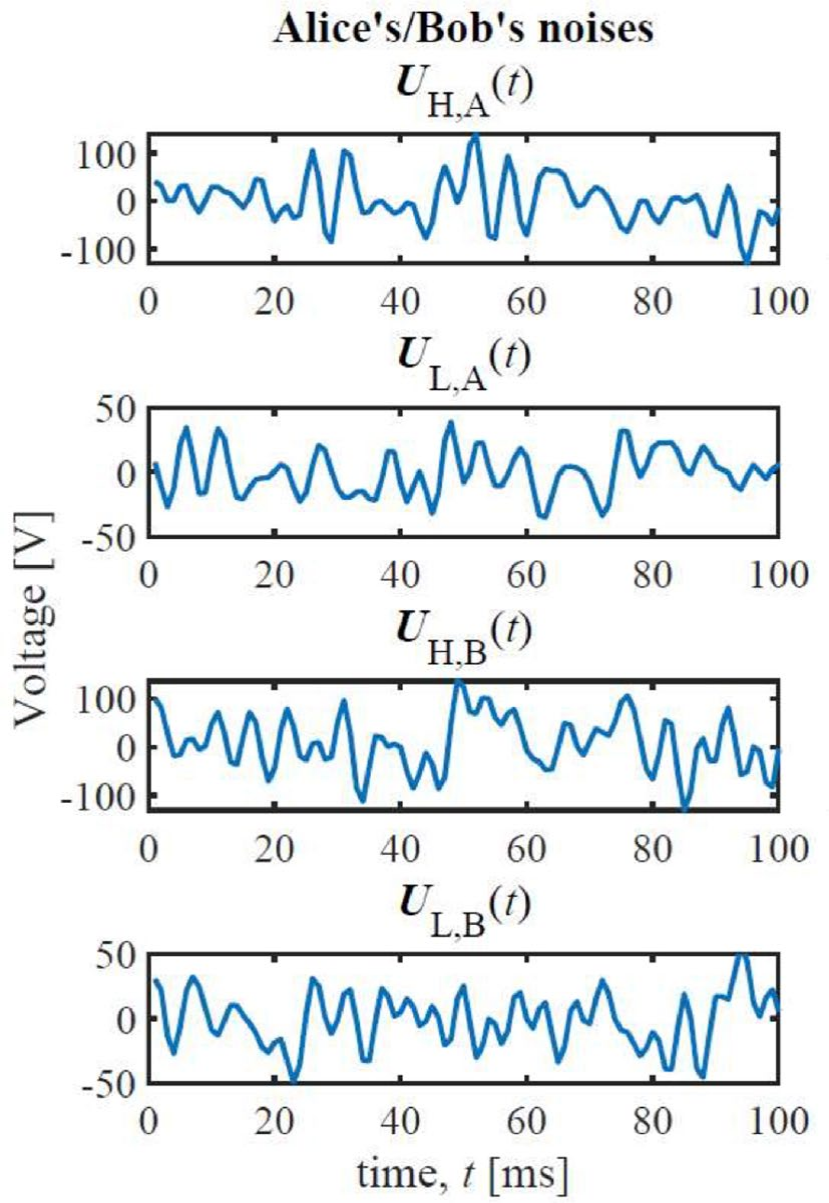
A realization of $U_{\textrm{H,A}}(t)$, $U_{\textrm{L,A}}(t)$, $U_{\textrm{H,B}}(t)$, and $U_{\textrm{H,B}}(t)$ (see Fig. 2) for Alice and Bob, displayed over 100 milliseconds^17–19,37^.

A realization of Alice’s and Bob’s noise voltages over 100 milliseconds is displayed in Fig. 6. $U_{\textrm{H,A}}(t)$ is the noise voltage of Alice’s $R_{\textrm{H}}$, $U_{\textrm{L,A}}(t)$ is the noise voltage of Alice’s $R_{\textrm{L}}(t)$, $U_{\textrm{H,B}}(t)$ is the noise voltage of Bob’s $R_{\textrm{H}}(t)$, and $U_{\textrm{L,B}}(t)$ is the noise voltage of Bob’s $R_{\textrm{L}}(t)$ (see Fig. 2). Each time step is one millisecond^17–19,37^.

#### Bit exchange

To emulate resistor selection by Alice and Bob during each BEP, we used the MATLAB randi function to draw independent binary values for each party. A value of 0 was mapped to $R_{\textrm{L}}$ and a value of 1 to $R_{\textrm{H}}$. When both parties chose the same resistor (HH or LL cases), the corresponding bit was treated as insecure and discarded. When the parties selected different resistors (HL or LH cases), the bit was accepted as secure and retained for the key, since an eavesdropper cannot distinguish between these two configurations based on the observed mean-square noise voltage.

Using this procedure, we simulated repeated BEPs until a key of length $L = 256$ bits was obtained (i.e., 256 bit exchanges). The resulting binary key was then converted to a hexadecimal representation of length $\frac{L}{4}= 64$ characters for use in the decentralized identity pipeline.

### DID setup

3We implemented the decentralized identity layer using the Veramo framework, which provides modular, open-source components for creating and managing DIDs and verifiable credentials^35,36^. Veramo’s plugin-based architecture allowed us to integrate a custom key management workflow while reusing standardized DID resolution and credential interfaces. Conceptually, our integration consists of three steps: (i) ingesting a KLJN-generated 256-bit binary key and converting it to a 64-character hexadecimal string, (ii) registering this hexadecimal key as the primary key material in Veramo’s key management system, and (iii) using Veramo’s DID provider for Ethereum (ethr-did) to derive and publish a corresponding DID document on the target network. Transport-level security between the DID agent and backend services is handled using TLS via OpenSSL libraries, ensuring that message exchange is cryptographically protected in addition to the physical-layer key generation.

To harden transport security between components, we coupled the Veramo agent with OpenSSL libraries, following configuration patterns from the official OpenSSL repository. This ensured that communication between the web server, DID agent, and backing services remained protected by widely-used TLS mechanisms in addition to the KLJN-based key generation^32^.

The practical steps to deploy the demonstrator involved setting up a Node.js application, installing the required Veramo packages (via yarn), configuring an agent with Ethereum-compatible DID providers, and wiring the KLJN-derived hexadecimal key into Veramo’s key management system. The overall DID lifecycle and credential issuance flow build on our earlier Web 3.0 DID prototype in, where further implementation details are available, but are extended here to accept physically generated keys from the KLJN scheme.

### Key integration

**Fig. 7:**
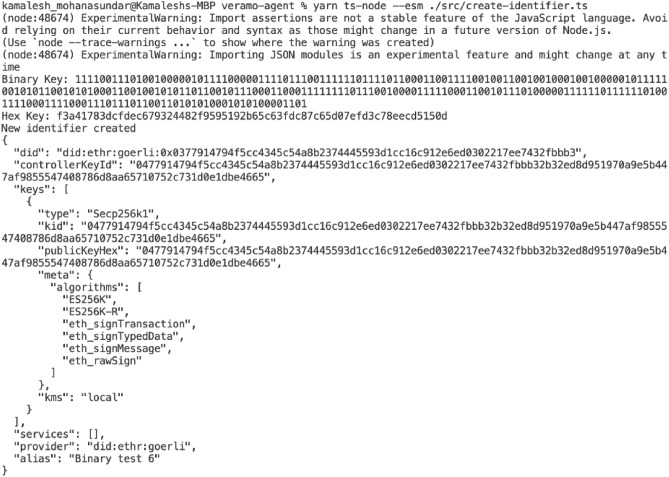
Generated DID with KLJN-derived key: shows the hexadecimal key representation, key identifier, algorithm type (ES256K), and Ethereum signing functions.

In order for Veramo to be able to properly read the KLJN-generated key, we created the “binaryToHex” function to regroup every four binary digits into a single hexadecimal character. Then, we input the resulting hex key into Veramo’s Key Management System, and we created an identifier by entering the command “yarn ts-node –esm ./src/create-identifier.ts”^2^. An example of a resulting DID is displayed in Fig. 7. The identifier has a unique DID string that is associated with a key pair. The identifier also contains Ethereum-specific signing functions such as “ES256K”, “ES256K-R” , and “eth_signTransaction that define how the keys can be used for transactions and interactions on the Ethereum blockchain.

### Randomness and statistical validation

To rigorously evaluate the statistical properties of KLJN-generated keys, we followed the methodology recommended in NIST SP 800-22 Rev. 1a^124^. We generated $N=100$ independent binary sequences, each of length $10^6$ bits, using the KLJN simulation described in the Methodology section. For each sequence we applied the full set of 15 NIST SP 800-22 tests at a significance level $\alpha = 0.01$.

For each test, we computed (i) the proportion of sequences whose individual p-values exceeded 0.01 and (ii) a second-level p-value assessing the uniformity of the p-value distribution across sequences, as prescribed in SP 800-22. The acceptable interval for the passing proportion was taken as $(1-\alpha )\pm 3\sqrt{\frac{\alpha (1-\alpha )}{N}}$, following NIST’s recommendation^124^. All tests produced passing proportions within this interval, and all second-level p-values exceeded 0.0001, indicating no statistically significant deviation from the ideal uniform distribution of p-values.

Table 1 summarizes the results across all 15 tests. These findings show that long KLJN-generated bit streams satisfy the standard statistical criteria for cryptographic-grade randomness, complementing the information-theoretic security guarantees derived from the physical model.

**Table 1:** Summary of cryptographic randomness tests applied to 256-bit keys generated by the KLJN scheme. All tests passed within acceptable p-value thresholds indicating high-quality randomness.

NIST Test	Sequences Passing / N	Passing Proportion	Expected Interval	Second-Level p-Value
Frequency (monobit)	98/100	0.98	0.96-1.00	0.43
Block Frequency	97/100	0.97	0.96-1.00	0.51
Runs	99/100	0.99	0.96-1.00	0.37
Longest Run of Ones	96/100	0.96	0.96-1.00	0.62
Rank	97/100	0.97	0.96-1.00	0.48
FFT	98/100	0.98	0.96-1.00	0.55
Non-Overlapping Templates	96/100	0.96	0.96-1.00	0.41
Overlapping Templates	97/100	0.97	0.96-1.00	0.39
Universal	98/100	0.98	0.96-1.00	0.34
Approximate Entropy	97/100	0.97	0.96-1.00	0.47
Cumulative Sums	98/100	0.98	0.96-1.00	0.52
Random Excursions	96/100	0.96	0.96-1.00	0.58
Random Excursions Variant	97/100	0.97	0.96-1.00	0.45
Serial	98/100	0.98	0.96-1.00	0.49
Linear Complexity	97/100	0.97	0.96-1.00	0.53

Our results demonstrate that the long KLJN-generated bit streams generated by the KLJN scheme consistently pass all critical tests within these suites, confirming their suitability for cryptographic applications. This statistical validation reinforces the physical security guarantees by confirming the randomness quality of the generated keys.

### Performance comparison

The KLJN scheme exhibits key generation rates that typically range from several hundred bits per second to tens of kilobits per second depending on system configuration and component quality^51^. Its core operation leverages elementary electronic components (e.g., resistors, switches) that are inherently low-cost and readily integrable into simple hardware modules. This results in negligible computational overhead and energy consumption compared to conventional cryptographic methods.

In contrast, elliptic curve cryptography (ECC) relies on computationally intensive mathematical operations such as scalar multiplication, point addition, and doubling on elliptic curve points. These operations generally require dedicated processors or specialized hardware like FPGAs, consuming considerable power and increasing latency^125–131^. While ECC is more efficient than older public-key approaches, its energy consumption remains significant enough to impact resource-constrained devices such as IoT endpoints or embedded systems. Consequently, the KLJN method offers a distinct advantage in scenarios demanding both strong information-theoretic security and low energy footprint.

This distinction is particularly critical in decentralized identity systems, where devices may possess limited processing capabilities and operate in energy-sensitive environments.

## Conclusion

Decentralized identity ecosystems depend on secure keys to function. Such keys are represented in hexadecimal, and they rely on computational algorithms for production. We showed that such keys can be generated in the absence of a computer, i.e. with statistical physical means. Since the keys are truly random, they are immune to vulnerabilities involving computational knowledge. We can then employ a base conversion algorithm and use the output to create a DID that was appended to an Ethereum blockchain.

This work was carried out on a local client-server network for a single user. Future work would involve integration on a cloud server and mass scalability of the KLJN scheme to make it accessible for multiple users.

## Data Availability

Data is provided within the manuscript. Original files are available upon request. Christiana Chamon Garcia (ccgarcia@vt.edu) is the point of contact for requesting data.

## Footnotes

1 This procedure is adapted from^1–3^, where it was originally described in detail.

2 This Johnson-noise emulation procedure follows the method originally described in^3^, with parameter values adjusted for the present study.

3 The example DID document structure shown in Fig. 1 follows the format and fields specified by the W3C DID Core standard and the ethr-did reference implementation, and was adapted from the public documentation in^2^.
